# Small-molecule amines: a big role in the regulation of bone homeostasis

**DOI:** 10.1038/s41413-023-00262-z

**Published:** 2023-07-24

**Authors:** Qian Zhang, Jirong Yang, Nan Hu, Juan Liu, Huan Yu, Haobo Pan, Di Chen, Changshun Ruan

**Affiliations:** 1 Research Center for Human Tissue and Organs Degeneration, Institute of Biomedicine and Biotechnology, Shenzhen Institute of Advanced Technology, Chinese Academy of Sciences, Shenzhen, 518055 China; 2 University of Chinese Academy of Sciences, Beijing, 100049 China; 3 Department of Nephrology, Shenzhen People’s Hospital (The Second Clinical Medical College, Jinan University; The First Affiliated Hospital, Southern University of Science and Technology), Shenzhen, 518020 Guangdong China; 4 Research Center for Computer-Aided Drug Discovery, Shenzhen Institute of Advanced Technology, Chinese Academy of Sciences, Shenzhen, 518055 China; 5 Shenzhen Healthemes Biotechnology Co., Ltd., Shenzhen, 518102 China; 6 Faculty of Pharmaceutical Sciences, Shenzhen Institute of Advanced Technology, Chinese Academy of Sciences, Shenzhen, 518055 China

**Keywords:** Homeostasis, Osteoporosis

## Abstract

Numerous small-molecule amines (SMAs) play critical roles in maintaining bone homeostasis and promoting bone regeneration regardless of whether they are applied as drugs or biomaterials. On the one hand, SMAs promote bone formation or inhibit bone resorption through the regulation of key molecular signaling pathways in osteoblasts/osteoclasts; on the other hand, owing to their alkaline properties as well as their antioxidant and anti-inflammatory features, most SMAs create a favorable microenvironment for bone homeostasis. However, due to a lack of information on their structure/bioactivity and underlying mechanisms of action, certain SMAs cannot be developed into drugs or biomaterials for bone disease treatment. In this review, we thoroughly summarize the current understanding of SMA effects on bone homeostasis, including descriptions of their classifications, biochemical features, recent research advances in bone biology and related regulatory mechanisms in bone regeneration. In addition, we discuss the challenges and prospects of SMA translational research.

## Introduction

**Fig. 1:**
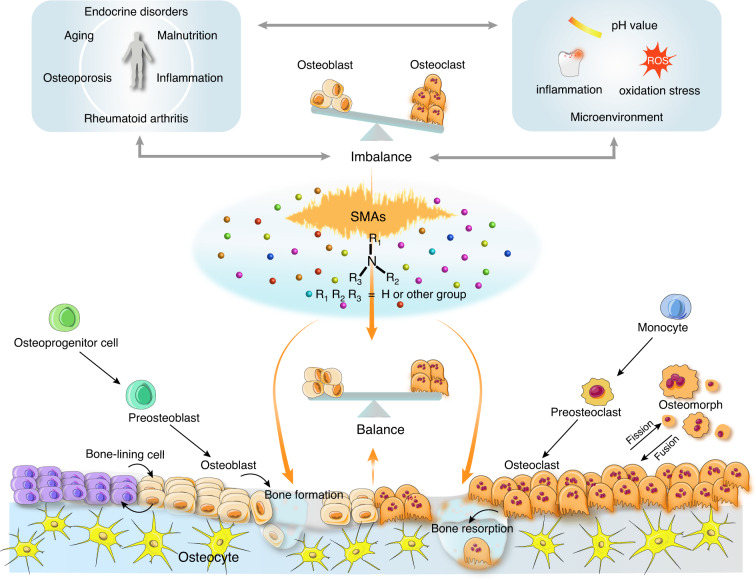
The roles of SMAs in the regulation of bone hemostasis. SMAs play a positive role in bone hemostasis through the regulation of bone formation, bone resorption, and the microenvironment. Factors such as aging, inflammation, malnutrition, or endocrine disorders and diseases such as osteoporosis and rheumatoid arthritis are often related to aberrant bone homeostasis, and factors some interact with microenvironments, including pH, inflammation, and oxidation stress factors. Factors, diseases, and microenvironments can lead to an imbalance between OBs and OCs

Bone is a vital organ in the body, providing support and protection for soft tissues and internal organs, and is involved in many biofunctions, e.g., hematopoiesis, mineral storage, endocrine regulation, and immunological modulation. Normal bone metabolism maintains bone homeostasis. However, many factors, such as aging, inflammation, malnutrition, or endocrine disorders, may interfere with the balance between bone formation and resorption, causing excessive bone resorption and bone loss.^1–4^ In addition, typical bone diseases such as osteoporosis, osteoarthritis (OA), and rheumatoid arthritis (RA) are often related to defects in bone remodeling (Fig. 1).^5,6^ Therefore, it is essential to restore the normal function of bone and reverse any imbalance in bone homeostasis through effective strategies.

Osteoblasts and osteoclasts are two principal cell types in bone tissue that play fundamental roles in maintaining bone homeostasis (Fig. 1). Osteoblasts (OBs) are the cells critical for bone formation. Mesenchymal stem cells (MSCs) and skeletal stem cells (SSCs) can differentiate into preosteoblasts (preOBs) and ultimately form mature OBs,^7^ which can be embedded in lacunae of a mineralized matrix and become osteocytes with a stellate shape.^8^ In contrast, osteoclasts (OCs) are multinucleated cells derived from hematopoietic stem cells (HSCs)^9^ and are critical for bone resorption. The preosteoclasts (pre-OCs) generated by HSCs migrate to the bone surface and fuse to form multinucleated OCs.^10–12^ The coordination and balance between OBs and OCs strictly control bone homeostasis. For instance, both macrophage-colony stimulating factor (M-CSF) and receptor activator for nuclear factor-κB ligand (RANKL) are key regulators that initiate the differentiation of pre-OCs into mature OCs.^13–16^ In addition, OC undergo fission to generate daughter cells named osteomorphs, which can be fused into OCs and thus recycled via the activation of RANKL.^17^ Furthermore, OBs and OCs can directly interact through membrane-bound mediators or secreted factors (Fig. 1). However, bone homeostasis can be disrupted when OB formation and activity are reduced and/or when bone resorption, such as that induced by inflammation, oxidation stress, or altered pH, is excessive.^18–20^ Therefore, strategies to regulate key molecular signaling pathways or to create favorable microenvironments for promoting OB formation/activity and/or inhibiting OC formation/activity need to maintain bone homeostasis. Recently, two major approaches have been taken for treating bone-related diseases by ameliorating bone homeostasis and bone regeneration: drug therapy and biomaterial-based repair. The former is used to regulate the key molecular signaling pathways for the maintenance of bone homeostasis, and the latter is mainly used to rebuild the local microenvironment to promote bone regeneration in defective bone.

Small-molecule amines (SMAs), nitrogen-containing compounds with a molecular weight below 900 daltons,^21^ are derived from a wide range of sources, including plants, animals, and microorganisms, or are artificially synthesized. Recently, extensive studies have demonstrated that SMAs, such as deferoxamine,^22^ dopamine,^23^ berberine,^24^ tetramethylpyrazine,^25^ and SB242784,^26^ show an excellent ability to maintain bone homeostasis via the regulation of molecular signaling pathway activity. Some SMAs, such as tofacitinib,^27,28^ baricitinib,^27^ and bortezomib,^29^ have been developed as drugs to treat bone diseases. Some SMA drugs that are used to treat nonbone diseases have also been found to exhibit potential efficacy in treating bone diseases. For example, metformin^30^ and glimepiride,^31^ used to treat type 2 diabetes mellitus, have shown positive effects on the attenuation of osteoporosis. Furthermore, the hydrogen atoms (one, two, or three) of the ammonia group in SMAs endow these molecules with special characteristics (e.g., alkalinity and antioxidant), which can maintain bone homeostasis via the local reconstruction of the extracellular microenvironment. Some SMAs are alkaloids, belonging to a large and diverse chemical group with alkali-like properties and at least one nitrogen atom in a heterocyclic ring structure.^32^ Interestingly, it has been reported that most of these alkaloids regulate bone homeostasis by inhibiting OC activity,^33–35^ which may be partially due to their alkalinity. In addition, due to the hydrogen atom in ammonia groups, SMAs with high reactivity can be used as monomers to develop SMA-based biomaterials for repairing bone defects. Upon the degradation of SMA-containing biomaterials, SMAs are released and reconstitute the local microenvironment to maintain bone homeostasis via the regulation of inflammation, oxidation or pH. Therefore, SMAs are suitable for developing novel drugs or biomaterials to treat bone-related diseases. However, the lack of information on SMA structure/bioactivity and underlying mechanisms of action has profoundly restricted the application of SMAs for treating bone diseases. Thus, a comprehensive analysis of the current developments in SMAs that enable bone homeostasis regulation is an extremely urgent need.

Given the aforementioned facts, in this review we thoroughly summarize the current understanding of SMAs in bone homeostasis (Fig. 1). First, we define SMAs, describing in detail their structures, classifications, and biochemical features. Next, SMA-based drugs and biomaterials, two key applications of SMAs to the regulation of bone homeostasis, are specifically discussed. Subsequently, the possible effects and mechanisms of SMA action on bone cells and the microenvironment are extensively elaborated. Finally, we further discuss current challenges and prospects in promoting SMA translational research.

## Definition of SMAs

### Structure and classification

**Fig. 2:**
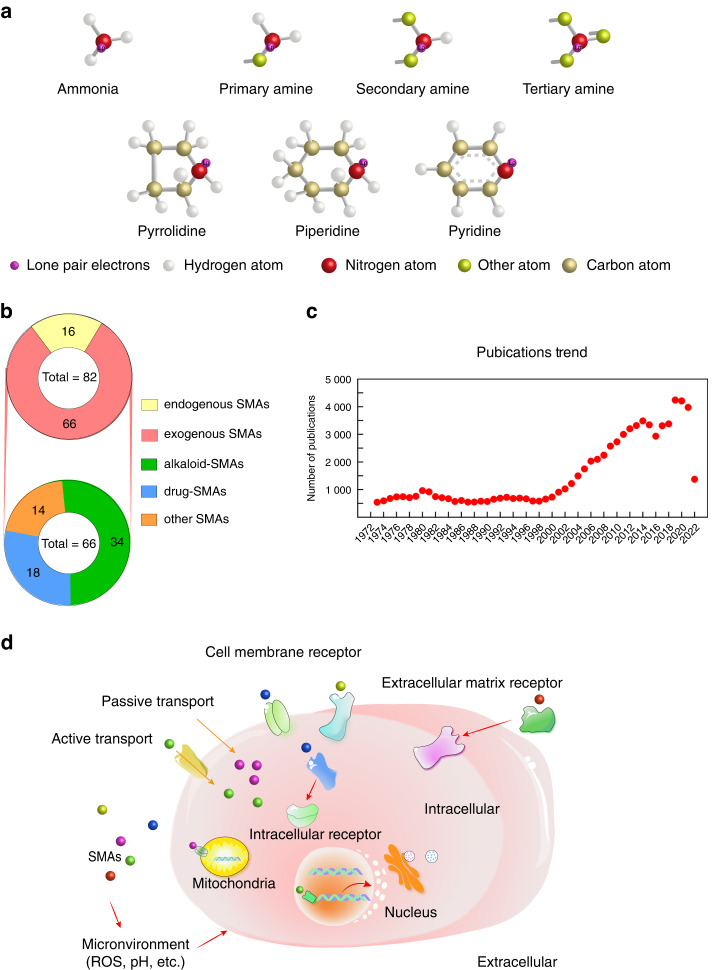
Literature analysis, classification, and mechanisms of SMA action. **a** Classification of amines. Amines are classified as primary, secondary, or tertiary ammonium compounds. Pyrrolidine and piperidine are secondary amines, and pyridine is a tertiary amine. **b** Classification of SMAs with osteotropic activity. Eighty-two kinds of SMAs associated with bone homeostasis include 16 endogenous and 66 exogenous SMAs. Exogenous SMAs are classified into three categories: alkaloid-SMAs, drug-SMAs (used as drugs in clinical applications), and other-SMAs. Data were obtained from the Web of Science using keywords (osteo- or bone) (amine/small molecular/alkaloid/drug) (until Jan 2022). **c** An analysis of the publications. Data were obtained from the Web of Science using keywords (osteo- or bone) and each name of the aforementioned 82 SMAs. **d** Possible approaches by which SMAs affect cells directly or indirectly. In a direct approach, SMAs bind to receptors on the cell surface or nuclear receptors inside cells or the mitochondrial membrane after entering cells through active or passive transport. In an indirect approach, cell behaviors are regulated by SMAs binding to extracellular matrix receptors, which further affects membrane proteins on the cell surface or influences the extracellular microenvironment, such as the pH value

SMAs, with a molecular weight of less than 900 Daltons, are generally classified as primary, secondary, or tertiary SMAs depending on the number of hydrogen atoms (one, two, or three) in the ammonia group, which is eventually replaced by organic groups (Fig. 2a). In chemical notation, SMAs can be categorized into three classes: RNH_2-_, R_2_NH-, and R_3_N-containing SMAs.^36^ In addition, some heterocyclic SMAs have been described (Fig. 2a). For instance, pyrrolidine and piperidine are five- and six-numbered heterocyclic secondary amine compounds.^36^ Pyridine is both an aromatic amine and a tertiary amine.

According to their sources, SMAs can be classified into endogenous SMAs and exogenous SMAs (Fig. 2b). In particular, endogenous SMAs, derived from the human body, usually play important roles in cell metabolism, mainly interacting with neurotransmitters (e.g., dopamine) and metabolic molecules (e.g., epinephrine and adenosine). Exogenous SMAs are derived from animals, plants, or microorganisms or are artificially synthesized amines, and herein, they are further classified into three categories: drug-SMAs, alkaloid-SMAs, and other-SMAs (Fig. 2b). Drug-SMAs are commercially available pharmaceuticals that have been used in clinical applications. Alkaloid-SMAs constitute a large and diverse group of chemicals with alkali-like properties that carry at least one nitrogen atom in a heterocyclic ring structure and are derived from a large variety of organisms, including bacteria, fungi, plants, and animals.

### Biochemical features

SMAs are show clear physiological activity, e.g., regulation of molecular signaling pathways or reconstruction of the extracellular microenvironment, due to their special biochemical features. First, SMAs show nucleophilic affinity for unbound electrons in nitrogen atoms, resulting in both the alkylation and acylation of an amine.^36^ Second, since lone pairs of electrons in nitrogen atoms tend to attract protons (namely, hydrogen ions), amines are often alkaline in nature. SMAs with alkalinity affect the pH of the extracellular microenvironment. In addition, SMAs exhibit antioxidative functions. Unbonded electrons in the nitrogen atoms of SMAs are easily removed and oxidized. Polyamines have been shown to inhibit lipid peroxidation in rat liver microsomes, scavenge free radicals and exert a powerful antioxidant effect in vivo, owing to the combination of anion- and cation-binding properties.^37^ The binding of polyamines to anions (phospholipid membranes and nucleic acids) contributes to a high local concentration at cellular sites particularly prone to oxidation, whereas the binding to cations efficiently prevents the site-specific generation of “active oxygen” (i.e., hydroxyl radicals and singlet oxygen). Spermidine has also been demonstrated to enhance the antioxidative activity of mung bean sprouts.^38^ Several previous studies have reported on the relationships between SMA chemical structures and specific biochemical features. However, the mechanisms of SMA actions in the regulation of key molecular signaling pathways and in the reconstruction of extracellular microenvironments (such as inflammation) need to be further clarified.

## State-of-the-art research on SMA functions in the regulation of bone homeostasis

### Recent progress

To understand the current status of SMAs in the regulation of bone homeostasis, we searched the Web of Science database with the following keywords: osteo- or bone and amine/small molecular/alkaloid/drug (before Jan. 2022). We found 83 SMAs with specific osteotropic activity (Fig. 2b) and then searched again using the keywords osteo- or bone and each name of these SMAs (before Jan. 2022). A total of 79 254 related documents published in the past 50 years were recovered, and they were analyzed in this project (Fig. 2c).

As shown in Fig. 2b, 82 kinds of bioactive SMAs involved in bone homeostasis were categorized into 16 different types of endogenous SMAs (listed in Table 1) and 66 different types of exogenous SMAs. Among the exogenous SMAs, 18 types of drug-SMAs (listed in Table 2), 34 types of alkaloid-SMAs (listed in Table 3), and 14 types of other-SMAs (listed in Table 4) were identified. An analysis of the aforementioned 79 254 publications displays (Fig. 2c) indicated that the number of SMA publications on bone homeostasis did not show an increasing trend until the early 21st century. The role of SMAs in bone homeostasis has attracted considerable attention, and research on SMAs in bone homeostasis is expected to surge in the future. Therefore, investigation into the effect and mechanism underlying SMA actions in bone homeostasis is of great interest.

**Table 1:** Endogenous SMAs and their molecular mechanisms of action

Name	Structural formula	Function	Cell types	Mechanism	References
Dopamine		OC↓ OB↑	hBMSCs; human CD14^+^ cell-derived OC precursor cells; PDLSCs; MC3T3-E1 preosteoblasts; RAW 264.7 cells	Activates ERK1/2, integrin α5/β1 and PI3K signaling; inhibits the cAMP/PKA/CREB pathway	^23,67,225–227^
Epinephrine		OB↑	ST2 cells; C2C12 cells	Activates BMP and cAMP/PKA signaling	^176^
Melatonin		OC↓ OB↑ inflammation ↓ oxidative stress ↓	RAW264.7 cells; DPSCs; MC3T3-E1 cells; hBMSCs	Activates p38/ERK signaling, the AMPK and STIM1/ ORAI1 pathways; inhibits MAPK and NFATc1 signaling	^73,228–233^
Adenosine		OC↓ OB↑	BMMs (C57BL/6 mice); MSCs; MC3T3-E1 cells	The A2B receptor inhibits the ERK1/2, p38 and NF-κB pathways (OCs); The A2B receptor and A(2A) receptor activates the Akt and Wnt pathways (OBs)	^96,97,234–236^
Glutamine		OB↑	HDPCs; ST2 cells; BMSCs; etc.	Activates BMP, Wnt, MAPK, and other pathways	^70,82,124^
Glutamic acid		OB↑	RAW264.7 cells; hFOB1.19 cells; MSCs (rat)	Activates Gln signaling	^83,237^
Taurine		OC↓ OB↑	RAW264.7 cells; the MLO-Y4 cell line (an osteocyte line); the IDG-SW3 cell line (an osteocyte line); MC3T3-E1 cells; MG63	Activates ERK and Wnt/β-catenin signaling pathways	^74,238–240^
Spermidine		OC↓ OB↑ inflammation ↓ oxidative stress ↓	chondrocytes (osteoarthritis patient); OCs (Std-ddY mice); RAW264.7 cells; human FLS cells	Inhibits the NF-κB pathway	^161,184,241,242^
Spermine		OC↓ OB↑	OCs (Std-ddY mice); RAW264.7 cells; hASCs	Inhibits +NF-κB pathway; activates β-catenin	^161,243^
Glucosamine		OB↑	Chondrocytes (SD rats); hFOB1.19 cells	Inhibits the mTOR (OB) pathway; promotes the Wnt (chondrocyte) pathway	^117,118^
Acetyl choline		OC↓	Pre-OCs (murine)	(nAChRs) Inhibits c-fos and NFATc1 signaling	^244^
Betaine		OB↑	hOBs; hBMSCs	Activate ERK IGF‑I and Ca2^+^-/calmodulin-dependent kinase II signaling; Inhibit mTOR pathway	^75,245^
Folic acid		OC↓ OB↑	hMSCs	N/A	^246,247^
Pyrroloquinoline quinone		OC↓ OB↑ oxidative stress ↓	MSCs (female C57/BL6J mice)	Inhibit ROS and NF-κB signaling	^210,248^
Sphingosine-1-phosphate		OB↑	C3H10T1/2 pluripotent stem cells; SaOS-2 cells; MC3T3-E1 cells	Activate PI3K/Akt and Wnt pathways	^100,249^
γ-Aminobutyric acid (GABA)		OB↑	hMSCs; RAW 264.7 cells	Activate TNFAIP3 signaling	^122^

**Table 2:** Drug-SMAs and their molecular mechanisms of action

Name	Clinical application	In vitro	In vivo	Function	Mechanism	Ref.
Deferoxamine	Acute iron poisoning	BMMs (C57BL/6 mice); MSCs	GIOP	OC↓ OB↑ angiogenesis ↑	Inhibits the electron transport chain and MAPK pathways; activates the HIF-1 and TGF-β1/Smad2 pathways	^115,138,250,251^
Metformin	Type 2 diabetes mellitus	PDLSC;s hEDT-SCs; MC3T3E1 cells; MSCs; ACSs; CV-MSCs	T2DM patients; OP; OVX	OC↓ OB↑	Inhibits RANKL; activates the AMPK, Wnt, Akt/Nrf2, BMP-4/Smad/Runx2 pathways	^121,206,252–257^
Benzydamine	Anti-inflammatory analgesics	BMMs (C57BL/6 mice)	OVX; LIOP	OC↓ OB↑ inflammation ↓	Inhibits NF-κB, ERK and p38 signaling	^45^
Cetirizine	Treatment of allergic conjunctivitis, etc.	N/A	CSE	OC↓	N/A	^46^
Cimetidine	Duodenal ulcer, gastric ulcer, reflux esophagitis, stress ulcer Zollinger–Ellison syndrome and chronic urticaria, etc.	BMMs (Lewis rat)	rat adjuvant arthritis	OC↓ inflammation ↓	Inhibits the histamine H2-receptor	^47^
Meclizine	Motion sickness	BMMs (C57BL/6 mice)	OVX	OC↓	Inhibits the NF-κB and MAPK pathways	^160^
Tranylcypromine	Depression	BMMs (C57BL/6 mice)	GIOP; OVX; LIOP	OC↓ OB↑	Inhibits the Akt and mTOR pathways; activates the BMP pathway	^119,258^
Piperazine	Roundworm and pinworm	MC3T3-E1 cells	N/A	OB↑	N/A	^58^
Doxycycline	Antibacterial and anti-infection agent	BM-MSCs	SIDR	OB↑	Activates the Wnt/β-catenin pathway	^48^
Enoxacin	Antibacterial and antibiotic	RAW 264.7 cells; primary marrow cells	N/A	OC↓	Inhibit ORV	^174^
Tofacitinib	Rheumatoid arthritis	MSCs	OVX; AM; adjuvant-induced arthritis	OC↓ OB↑ inflammation ↓	Inhibits JAK and RANKL signaling; activates the Wnt pathway	^27,28,259^
Baricitinib	Rheumatoid arthritis	MSCs; PMCCs (male ddY mice)	OVX; AM	OC↓ OB↑ inflammation ↓	Inhibits JAK and RANKL signaling; activates Wnt pathway	^27,45^
Benidipine	Essential hypertension	BMSCs	OVX	OB↑	Activates the Wnt/β-catenin pathway	^86^
Glimepiride	Type 2 diabetes mellitus	OB (Sprague–Dawley rats)	N/A	OB↑	Activates the PI3K/Akt/eNOS pathway	^31^
Bortezomib	Adult patients with multiple myeloma	BMMs (female ICR mice); osteoblast-derived UMR106-01 cells; POBs	OVX; FRIO; AIA	OC↓ OB↑ inflammation ↓	Inhibits the proteasome and NF-κB pathway	^29,40,260^
Odanacatib	Osteoporosis (Phase III)	N/A	OVX monkeys; ROFR	OC↓ OB↑ inflammation ↓	Inhibits Cathepsin K	^261–263^
Saracatinib	Cancer, osteosarcoma, etc. (Phase II)	RAW 264.7 cells; PC-3 prostate cancer cells; rabbit OCs; fetal calvarial mouse OCs; hPBMCs	OLIP	OC↓	Inhibits the Src, NF-κB and p38 pathway	^41,42^
Dasatinib	Lymphoblastic or chronic myeloid leukemia	hBM-MSCs; hMSCs; MG-63; PBMCs	OIRA; CRMO	OC↓ OB↑ inflammation ↓	Inhibits Src, ERK1/2 pathway; activates the Wnt/β-catenin and Hippo-YAP pathways	^129,130,193^

**Table 3:** Alkaloid-SMAs and their molecular mechanisms of action

Name	Source	Cell types	Function	Mechanisms	In vivo	Ref.
Neferine	*Nelumbo nucifera* (lotus)	BMMs(C57BL/6 mice) ; MC3T3 E1	OC↓ OB↑	Inhibits the NF-κB pathway	OVX	^153^
Nuciferine	*Nelumbo nucifera* (Lotus)	BMMs(Balb/c mice)	OC↓	Inhibits the NF-κB and MAPK pathways	OVX; BCIO	^151,152^
Tetramethylpyrazine	*Ligusticum chuanxiong* Hort.	BMSCs; hPDLSCs; human chondrocytes; and human osteosarcoma cells	OC↓ OB↑	Inhibits the NF-κB and mTOR pathways; inhibits RANKL and IL-6 signaling; activates the AMPK pathway	GIOP	^25,131,186,187,264^
Tomatidine	*Solanaceae*	BMMs (C57BL/6 mice)	OC↓ inflammation ↓	Inhibits the NF-κB pathway	OVX	^154^
Neotuberostemonine	*Stemona tuberosa*	BMMs (C57BL/6 mice)	OC↓	Inhibits the NF-κB pathway	—	^155^
Tetrandrine	*Stephania tetrandra* S. Moore	BMMs (C57BL/6 mice)	OC↓ inflammation ↓	Inhibits the NF-κB, PI3K/AKT, and MAPK pathways	OVX; SNM; TiP-IMAPO	^156–158^
Berberine	*Berberis* Linn.	MC3T3-E1 cells, hMSCs, RAW264.7 cells, etc.	OC↓ OB↑ inflammation ↓ oxidative stress ↓	Inhibits the NF-κB, PI3K/Akt and NFAT pathways; activates the PKA, p38 MAPK, Wnt/β-catenin and AMPK pathways	SO(SAMP6); OVX; LIOP; GIOP	^188,265,266^
Sanguinarine	*Sanguinaria Canadensis*	BMMs, RAW264.7 cells, MC3T3-E1 cells	OC↓	Inhibits the NF-κB and ERK pathways; activates the AMPK/Smad1 pathway	OVX	^106,136^
Vinpocetine	*Catharanthus roseus* (L.) G. Don, Gen. Hist	BMMs (C57BL/6 mice)	OC↓ inflammation ↓ oxidative stress ↓	Inhibits the MAPK and NF-κB pathways	OVX	^33^
Norisoboldine	*Lindera aggregata* (Sims) Kosterm.	BMMs (male ICR mice); RAW264.7 cells	OC↓	Inhibits the MAPKs/NF-κB/c-Fos/NFATc1 signaling and HIF pathways	OVX	^139,159^
Aconine	*Aconitum carmichaelii* Debx.	RAW264.7 cells	OC↓	Inhibits the NF-κB pathway	-	^35^
Evodiamine	*Tetradium ruticarpum* (A.Jussieu) T. G. Hartley	BMMs (C57BL/6 mice)	OC↓	Inhibits the NF-κB, MAPK, and calcium pathways	OVX; GIOP (zebrafish)	^34,71^
Rutaecarpine	*Tetradium ruticarpum* (A.Jussieu) T.G.Hartley	BMMs	OC↓	Inhibits the NF-κB and NFATc1 pathways	-	^267^
Stachydrine	*Leonurus heterophyllus* Sweet	BMMs (C57BL/6 mice)	OC↓	Inhibits the NF-κB and Akt pathways	LIOP	^164^
Dauricine	*Menispermum dauricum* DC.	BMMs (C57BL/6 mice)	OC↓ oxidative stress ↓	Inhibits ROS/PP2A/NF-κB signaling	LIOP	^189^
Cytisine	*Leguminosae* (Fabaceae)	BMMs (C57BL/6 mice); RAW264.7 cells; BMSCs(C57BL/6)	OC↓	Inhibits the JNK/ERK/p38-MAPK, IκB alpha/p65-NF-κB and PI3K/AKT pathways	OVX	^140^
l-Tetrahydropalmatine	*Corydalis* DC	BMMs (C57BL/6 mice); RAW264.7 cells	OC↓	Inhibits the NF-κB and MAPKs pathways	OVX	^141^
Arecoline	*Areca catechu* Linn.	BMMs (C57BL/6 mice); MC3T3-E1 cells	OC↓ OB↑	Inhibits RANKL, NFATc1, and c-Fos signaling	LIOP	^132^
Piperine	*Piper nigrum* Linn.	RAW264.7 cells; MC3T3-E1 cells	OC↓ OB↑	Inhibits p38/c-Fos/NFATc1 signaling; activates the Wnt and AMPK pathways	OVX	^81,105,137^
Lycorine	*Lycoris radiata* (L’Her.) Herb.	BMMs (C57BL/6 mice)	OC↓	Inhibits mROS/TRPML1/TFEB signaling; inhibits the MAPK pathway	OVX; TiP-IMAPO; LIOP	^268,269^
Sinomenine	*Sinomenium acutum* Rhed. et Wils.	RAW264.7	OC↓	Activates caspase-3 activity; downregulates IL-8/CXCR1, TLR4/TRAF6 and c-Fos/NFATc1 signaling	LIOP; BCIO	^270–272^
Diaporisoindole E	Diaporthe sp. SYSU-HQ3	BMMs (C57BL/6 mice)	OC↓	Inhibits the PI3K/AKT and MAPK pathways	-	^167^
Cinchonine	*Cinchona ledgeriana* (Howard) Moens ex Trim.	BMMs (C57BL/6 mice); RAW264.7 cells; BMSCs	OC↓ OB↑	Inhibits the NF‐κB pathway	OVX; LIOP	^133^
Aminocoumarins	*Streptomyces*	BMMs (male ICR mice)	OC↓	Inhibits the MAPK pathway; inhibits IL-1β, TNF-α, and iNOS signaling	LIOP	^273^
Nitensidine A	*Pterogyne nitens*	BMMs (male ddY mice)	OC↓	-	—	^190^
Theobromine	*Theobroma cacao* L.	hMSCs	OB↑	-	Bone development (SD rats) during pregnancy, lactation, and the early growth period	^274^
Leonurine	*Leonurus artemisia*	RAW 264.7 cells; BMMs; MC3T3-E1 cells	OC↓ OB↑	Inhibits the NF-κB and PI3K/Akt pathways; activates the PI3K/Akt/mTOR pathway	OVX	^80,275^
Harmine	*Peganum harmala* L.	BMMs (male ddY mice); RAW264.7 cells;UAMS-32 cells; MC3T3-E1 cells	OC↓ OB↑	Inhibits c-Fos and NFAT c1 signaling; activates the BMP and Runx2 pathways	OVX	^120,276^
Vindoline	*Catharanthus roseus* (L.) G. Don	BMMs (C57BL/6 mice)	OC↓ oxidative stress ↓	Inhibits the MAPK pathway	OVX	^209^
Matrine and its derivatives	*Sophora flavescens* Alt.	BMSCs (rats)	OC↓ OB↑	Inhibits the NF-κB, AKT, and MAPKs pathways	OVX; RMEM	^277–279^
Securinine	*Flueggea suffruticosa* (Pall.) Baill.	BMMs (male ICR mice)	OC↓	Inhibits the p38, Akt, JNK, IκB, c-Fos, and NFATc1 pathways	LIOP	^280^
Magnoflorine	Magnolia or Aristolochia	BMMs (C57BL/6 mice)	OC↓	Inhibits the MAPK and NF-κB pathways	Ti particle-induced mice air pouch osteolysis	^281^
Nitidine chloride	*Zanthoxylum nitidum* (Rutacease) and *Fagara zanthoxyloides*	BMMs (C57BL/6 mice)	OC↓	Inhibits the RANKL-induced NF-κB and NFATc1 pathways	OVX	^282^
Largazole	a cyanobacterium in the genus *Symploca*	C2C12 cells	OB↑	Activates the Runx2 and BMPs pathways	Mouse calvarial bone formation assay; rabbit calvarial bone fracture healing model	^283,284^

**Table 4:** Other SMAs and the molecular mechanism

Name	In vitro	In vivo	Function	Pathways	References
OSU53	OB-6	N/A	oxidative stress↓(OB)	Activates the AMPK pathway	^108^
AICAR	hFOB1.19 cells; hBMCs	N/A	oxidative stress↓(OB)	Activates the AMPK and ERK pathways	^109^
A-769662	MG-63 cells; MC3T3-E1 cells	N/A	oxidative stress↓(OB)	Activates the AMPK pathway	^110^
GSK621	MC3T3-E1; RAW 264.7; BMMs (C57BL/6 mice); PBMCs	N/A	oxidative stress↓(OB)	Activates the AMPK pathway	^111,112^
Compound 13	murine calvariae osteoblasts	N/A	oxidative stress↓(OB)	Activates the AMPK pathway	^207^
SB242784	human osteoclasts	OVX (rats); RIHT (rats)	OC↓ bone resorption ↓	Inhibits ORV	^26,171,285^
FR167356	calvariae cells (Wistar rats)	OVX (rats); RIHT (rats)	OC↓ bone resorption ↓	Inhibits ORV	^286^
FR202126	calvariae cells (Wistar rats)	OVX (rats); RIHT (rats)	OC↓ bone resorption ↓ inflammation ↓	Inhibits ORV	^287,288^
FR177995	mouse calvariae cells	AIA	OC↓ bone resorption ↓ inflammation ↓	Inhibits ORV	^289^
KM91104	BMMs (C57BL/6 mice); RAW264.7 cells	N/A	OC↓	Inhibits ORV	^173^
KMUP-1	MC3T3-E1 cells	N/A	OB↑	Activates BMP-2/Smad1/5/8, cAMP, cGMP signaling and the Wnt/β-catenin pathway	^290^
Fumitremorgin C	BMMs (C57BL/6 mice)	N/A	OC↓	Inhibits the NF-κB and MAPK pathways	^291^
SC-79	MC3T3-E1 cells	N/A	oxidative stress↓(OB)	Activates the Akt-Nrf2 pathway	^165^
6-Bromoindirubin-3’-oxime	BMSCs	N/A	OB↑	Activates the Wnt pathway	^88^

*OVX* ovariectomized osteoporosis, *BCIO* breast cancer-induced osteolysis, *GIOP* glucocorticoid-induced osteoporosis, *TiP-IMAPO* Ti particle-induced mouse air pouch osteolysis, *SNM* sciatic-neurectomized mice, *SOP* senile osteoporosis, *LIOP* lipopolysaccharide-induced osteoporosis, *RME* rapid maxillary expansion model, *ACSs* adipose-derived multipotent mesenchymal stem cells, *CV-MSCs* human chorionic villous mesenchymal stem cells, *hEDT-SCs* human exfoliated deciduous teeth stem cells, *AM* Arthritis model, *PMCCs* primary murine calvarial cells, *SIDR* streptozotocin-induced diabetic Wistar rat, *BM MSCs* bone marrow mesenchymal stromal cells, *BMSCs* bone marrow stromal cells, *PDLSCs* periodontal ligament stem cells, *ST2* BMP-2-responsive pluripotent murine bone marrow-derived stromal cells, *C2C12* pluripotent myoblasts, *hACS* human adipose-derived stem cells, *SD* rats Sprague‒Dawley rats, *PBMCs* peripheral blood mononuclear cells, *hOBs* human osteoblast‑like cells, POBs primary calvarial osteoblasts, *FRIO* focal radiation-induced osteoporosis, *AIA* adjuvant-induced arthritis, *ROFR* rabbit osteotomy model of fracture repair, *CSE* calvarial suture expansion, *OLIP* osteolytic lesions induced in a PC-3 bone model, *hPBMCs* human peripheral blood mononuclear cells, *OIRA* osteoporosis induced by retinoic acid, CRMO chronic recurrent multifocal osteomyelitis, *hBMCs* human bone marrow-derived cells, *RIHT* retinoid-induced hypercalcemia in thyroparathyroidectomized rats, *RMEM* rapid maxillary expansion model

In view of the beneficial effects of SMAs on bone homeostasis, it is necessary to try to identify the mechanisms of action at the cellular and molecular levels. As shown in Fig. 2d, SMAs may affect cellular function directly or indirectly. Through a direct mechanism, they bind to receptors on the cell surface or receptors inside the nucleus or on the mitochondrial membrane after entering cells through active or passive transport. Through an indirect mechanism, SMAs may regulate cellular behaviors by binding to extracellular matrix receptors, which further affect membrane proteins on the cell surface or influence the extracellular microenvironment, such as its pH and oxidative inflammatory status.

### SMA applications

#### SMA-based drugs

**Fig. 3:**
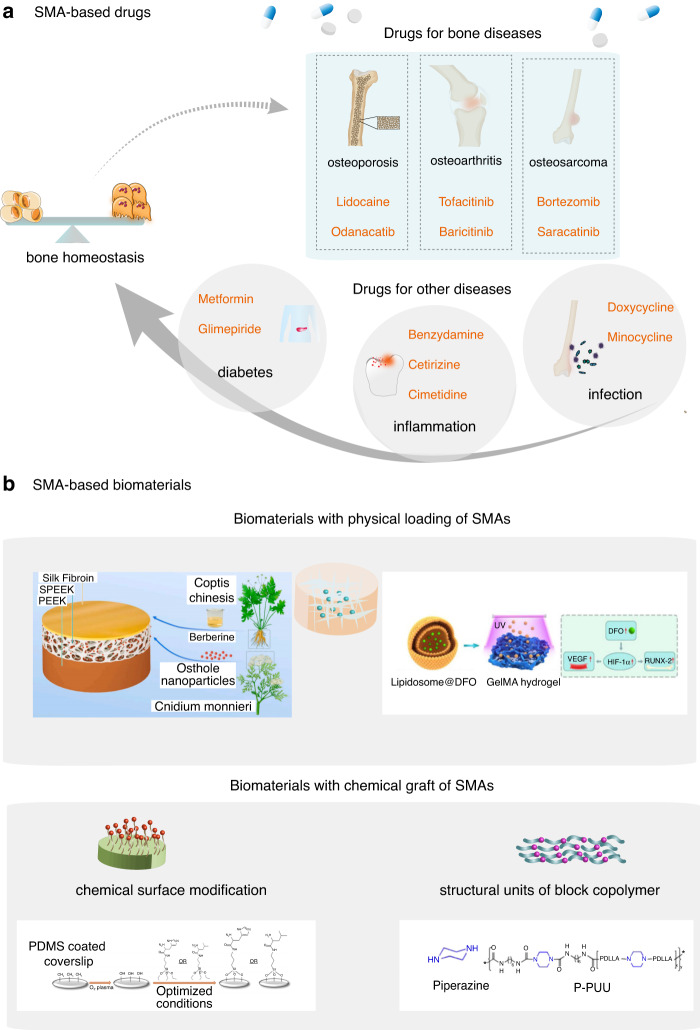
The main routes of SMA application for maintaining bone homeostasis. Two major routes of SMA applications are used for treating bone-related diseases by ameliorating bone homeostasis: **a** SMA-based drugs and **b** SMA-based biomaterials: SMA-based drugs, including drugs used for treating bone diseases and drugs for other diseases that show positive effects on bone homeostasis. SMA-based biomaterials include biomaterials on which SMAs have been physically loaded (for example, sponge-inspired sulfonated polyetheretherketone loaded with polydopamine-protected osthole nanoparticles and berberine;^52^ liposome carriers of desferrioxamine^53^) and biomaterials with chemical engrafting of SMAs (for example, polydimethylsiloxane surface with histidine- and leucine-conjugated (3-aminopropyl)-triethoxysilane;^56^ piperazine-based polyurethane-urea scaffolds^58^)

The use of SMAs as drugs (SMA-based drugs) is a vital application for bone homeostasis (Fig. 3a). The primary application of SMAs is for the treatment of osteoporosis, OA, RA, or tumors related to aberrant bone homeostasis. Phase III trials for Odanacatib, an inhibitor of cathepsin K, or the treatment of osteoporosis have been completed.^39^ Some drug SMAs have been developed to treat RA through the regulation of bone homeostasis. For instance, as Janus kinase (JAK) inhibitors, tofacitinib and baricitinib have been used to treat RA in the clinic. Recent studies have shown that they inhibit OC formation as well as promote OB formation.^27^ In addition, it has been reported that some drug-SMAs developed for the treatment of osteosarcoma show excellent osteotropic activity. Bortezomib is a proteasome inhibitor used to treat multiple myeloma and has been shown to promote bone formation in vivo.^29,40^ Similarly, had completed Phase II trials of saracatinib, an inhibitor of Src, for the treatment of osteosarcoma indicated that exhibited osteotropic activity.^41,42^

Since other diseases (e.g., diabetes and inflammation) often affect bone homeostasis, drug-SMAs used to treat these diseases might exert a positive effect on bone homeostasis^43^ (Table 2). Diabetes adversely affects bone homeostasis due to impaired glucose metabolism and toxic effects of glucose oxidation derivatives.^44^ Moreover, it has been reported that patients with diabetes present with lower bone quality and increased fracture risk compared with nondiabetic patients.^43^ Metformin and glimepiride, drugs used to treat type 2 diabetes mellitus, have been studied for the regulation of bone homeostasis. In particular, metformin has been demonstrated to inhibit OC formation and promote OB differentiation in vitro and in vivo; therefore, it might be used for the treatment of osteoporosis in the future.^30^ Additionally, numerous studies have indicated that metformin increased bone density and reduced bone turnover and fracture risk in patients with T2DM.^30^ As previously mentioned, inflammation can disrupt bone homeostasis. Therefore, some anti-inflammatory drug-SMAs such as benzydamine,^45^ cetirizine,^46^ and cimetidine^47^ display beneficial effects on the treatment of inflammation-related bone diseases. For example, benzydamine, a nonsteroidal anti-inflammatory drug, has been shown to prevent OC differentiation and inhibit interleukin-1β production.^45^ Cetirizine, a histamine 1 receptor antagonist, has been demonstrated to promote bone healing.^46^ In addition, other drug-SMAs with antibacterial or anti-infection functions, such as doxycycline,^48^ exert a positive influence on bone homeostasis (Table 2). For instance, Gomes, P. S., et al.^48^ declared that doxycycline, an antibacterial and anti-infection drug, restored the impaired osteogenic commitment of bone marrow mesenchymal stromal cells (BMSCs) derived from diabetic patients by activating Wnt/β-catenin signaling. Phase III trials to evaluate Lidocaine, a local anesthetic drug, for the treatment of postmenopausal osteoporosis and a Phase IV trial for its use as a treatment of knee and hand OA have been completed.^49^ Taken together, the usage of drugs used to treat other nonbone diseases may be expanded to applications to treat bone diseases.

#### SMA-based biomaterials

The use of SMA monomers to develop novel biomaterials (SMA-based biomaterials**)** is another pivotal way to increase SMA applications. Although most SMAs are still being evaluated through preclinical research or show no precise medicinal effects, many SMAs are used to prepare SMA-based biomaterials with enhanced osteotropic activity. Recently, two main types of SMA application via SMA-based biomaterials have been described (Fig. 3b): a) biomaterials with physically loaded SMAs and b) biomaterials with chemically engrafted SMAs, including chemical surface modifications and structural units of block copolymers.

##### Biomaterials after physical SMA loading

The method of the physical loading of bioactive SMAs involves SMA adsorption with biomaterials via weak interactions, such as hydrogen bonding, electrostatic attraction, and conjugation. The application of DA as a polydopamine (PDA) coating is the most common physically loaded modification due to its excellent conjugation effect. Li et al.^50^ reported that a PDA coating enhanced the attachment and proliferation of MC3T3-E1 cells to the surface of 3D-printed porous Ti6Al4V scaffolds and promoted the expression of osteogenic genes and proteins, making its use a great strategy for the orthopedic application of implants. In addition, berberine has become an important molecule for the physical modification of biomaterials via electrostatic attraction. Hu et al.^51^ fabricated a biomimetic CaP scaffold coating with berberine onto Ag nanoparticles loaded with silk fibroin. According to research by Sang et al., the modification of a polyether ether ketone (PEEK) surface with osthole particles and berberine led to effective osteogenic and antibacterial PEEK functions^52^ (Fig. 3b). Since the lifespan of deferoxamine (DFO) is extremely short, a sustained release system was needed. To meet demand, Chen et al.^53^ developed a novel drug delivery system by combining DFO-loaded liposomes carrying photocrosslinked gelatin hydrogel to control the sustained release of DFO from the hydrogel matrix (Fig. 3b). The results showed that DFO simultaneously released from the hydrogel facilitated angiogenesis and osteogenesis, further accelerating new vessel formation and bone regeneration.^53^ Song et al.^54^ fabricated titanium implants coated with doxycycline-loaded coaxial nanofibers and found that the implants enhanced osseointegration. In addition, some alkaloid-SMAs with osteotropic activity have been loaded into scaffolds. For instance, Wang et al.^55^ revealed that berberine-loaded porous calcium phosphate cements with the release of berberine sustained for as long as 9–10 days enhanced BMSC proliferation and differentiation, obviously increased the ALP and mineral deposition levels, and significantly promoted bone regeneration in osteoporotic rats. Physical loading of SMAs into biomaterials is a facile method and can reduce the amount of chemical residue. However, the release SMAs relatively quickly. To better control drug release, methods of chemically grafting SMAs into biomaterials have become increasingly attractive.

##### Biomaterials with chemical-engrafted SMAs

Two main ways to integrate SMAs with biomaterials independently are modification of the material surface with chemicals or structural units of block copolymers. A previous report disclosed that histidine-conjugated (3-aminopropyl)-triethoxysilane-modified polydimethylsiloxane led to higher ALP activity and greater deposition of mineralized ECM components than a control group of hFOBs^56^ (Fig. 3b). Compared with surface chemical modification, a greater number of biomaterials with SMAs added as structural units of block copolymers have been reported. Some simple SMAs, such as dopamine, lysine, and piperazine, can be polymerized to form copolymer biomaterials. Cui et al.^57^ successfully synthesized foamy poly(Nε-benzyl formateoxycarbonyl-L-Lysine) (PZL) and poly(Nε-benzyl formateoxycarbonyl-L-lysine-co-L-phenylalanine) (PZLP) scaffolds, and the results of analysis showed that PZL scaffolds increased the adhesion, proliferation and OB differentiation of MC3T3-E1 cells compared to the effects of PZLP scaffolds. Previous work from our laboratory revealed that a series of piperazine-based polyurethane-urea (P-PUU) modifications enhanced OB differentiation as the number of piperazine units was increased within a certain concentration range both in vitro and in vivo (Fig. 3b).^58^ Later, we found that piperazine itself regulated OB differentiation in a dose-dependent manner.^58^ In a recent study, Mao et al.^59^ synthesized a novel citrate-based biodegradable elastomeric poly(citric acid-1,8-octanediol–1,4-bis(2-hydroxyethyl) piperazine (BHEp)) (POPC) material by incorporating the alkaline fragment BHEp and then fabricated 3D printed POPC/β-tricalcium phosphate porous scaffolds (PTCPs). The results of a subsequent analysis demonstrated that PTCP neutralized the acidic microenvironment to enhance adhesion, proliferation, and bone regeneration owing to the activity of BHEp. Therefore, some SMAs can not only serve as a unit of a base material that enhances the physical properties of the treatment but can also promote bone formation after release. Primary amines in SMAs may be among the reasons for SMAs promotion of the proliferation and osteogenic differentiation of MSCs.

Chemical grafting of SMAs into biomaterials allows SMAs to directly affect the properties of a biomaterial itself. Surface chemical modification affects mainly surface properties, such as hydrophobicity, while the polymerization method affects the physicochemical properties of the whole biomaterial. In contrast, chemical combinations can better achieve slow drug release. However, although some problems with polymerized materials, such as degraded products of the SMA-based biomaterials, may not be evident for all SMA monomers, some chemical fragments can increase the complexity of the material for cellular action.

## Mechanisms of SMA action in the regulation of bone homeostasis

### Regulation of bone cell behaviors

#### Promotion of OB formation

**Fig. 4:**
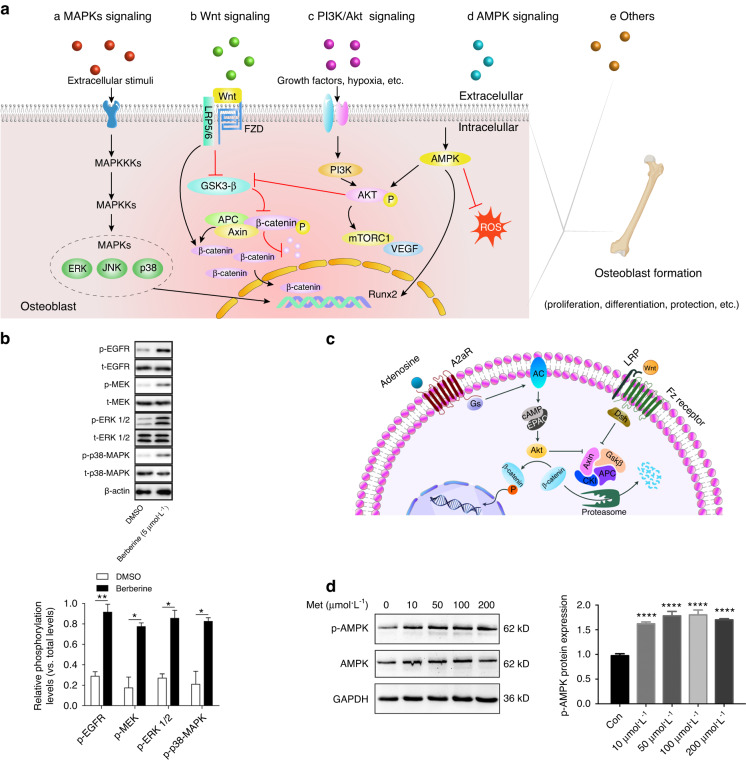
Mechanisms underlying the SMA promotion of osteoblast (OB) formation. **a** a) MAPK signaling pathway; b) Wnt signaling pathway. After stimulation of Wnt, the destruction complex of the β-catenin protein, the central molecule in canonical Wnt signaling, composed of GSK-3β, APC, and Axin1/Axin2, is dissociated, leading to the accumulation of β-catenin in the cytoplasm, and then, β-catenin is translocated to the nucleus, where it enhances the expression of its downstream target genes. c) PI3K/Akt signaling pathway. Growth factors or hypoxia activate PI3K and other factors, leading to the phosphorylation of AKT, which inactivates GSK3-β and riggers the mammalian target of rapamycin (mTOR) pathway, vascular endothelial growth factor (VEGF) activity, etc. d) AMPK signaling pathway. The activation of AMPK promotes osteogenic differentiation by upregulating the expression of Runx2 and suppressing the apoptosis of OBs induced by H_2_O_2_. e) Other pathways that affect bone formation. **b** Berberine activates the MAPK pathway.^69^ *P< 0.05, **P < 0.01. **c** The activation of the adenosine A2A receptor (A2AR) triggers Akt signaling and enhances the nuclear localization in β-catenin in OBs.^97^ **d** Effect of metformin treatment on the activation of AMPK in stem cells from human exfoliated deciduous teeth^107^

OBs, the chief bone-making cells with abundant mitochondria and a huge Golgi apparatus, synthesize a variety of extracellular matrix proteins, such as high levels of type I collagen (COLI), osteocalcin (OCN), alkaline phosphatase (ALP) and osteopontin (OPN), and subsequently promote mineralization through the deposition of calcium phosphate in the form of hydroxyapatite, the major inorganic component of bone.^60–63^ Therefore, the promotion of OB formation is crucial for bone homeostasis. Recent investigations have shown that certain SMAs positively regulated OBs through multiple signaling pathways, including the mitogen-activated protein kinase (MAPK) pathway, Wnt pathway, PI3K/Akt pathway, AMPK pathway, and mTOR pathway (Fig. 4a).

##### The MAPK signaling pathway

MAPKs are important mediators of a cell signaling pathway with activity that is regulated through a three-tiered cascade composed of MAPKs, MAPK kinases (MAPKKs, MKK or MEK) and MAPKK kinases or MEK kinases (MAPKKKs and MEKKs). Extracellular signal-regulated kinase (ERK), c-Jun N-terminal kinase (JNK), and p38 are family members of MAPKs that play significant roles in cell proliferation, differentiation, and apoptosis.^64^ ERK activation promoted osteogenic differentiation and bone formation by upregulating the expression of β-catenin and Runx2.^65,66^ Wang et al.^67^ demonstrated that a low concentration (5 nmol·L^−1^) of DA activated the D1 receptor and further promoted osteogenesis through the activation of the ERK signaling pathway mediated by enhanced Runx2 transcriptional activity, whereas blocking the ERK1/2 signaling pathway inhibited the dopamine-induced osteogenic differentiation of human bone mesenchymal stem cells (hBMSCs). Berberine has been shown to facilitate the proliferation of human dental pulp stem cells (hDPSCs) in a dose-dependent pattern and stimulate osteogenic differentiation partially by activating MAPK pathways^68,69^ (Fig. 4b). It has also been shown that glutamine promoted the proliferation, differentiation and migration of human dental pulp cells (hDPCs) by activating p38, JNK, and ERK, which were blocked by specific MAPK inhibitors, indicating that the MAPK pathway is one of the important signaling pathways in the induction of glutamine-mediated proliferation and differentiation of hDPCs.^70^ In addition, evodiamine might prevent osteoporosis by reversing an imbalance in bone formation/bone resorption and activating the MMP3-OPN-MAPK signaling pathway. Compared to those in the control group, the significant decrease in mRNA and protein levels related to MAPK activity and calcium deposition in dexamethasone-induced osteoporosis in zebrafish was reversed by evodiamine treatment.^71^ In addition, Kim et al.^72^ demonstrated that salicylideneamino-2-thiophenol enhanced the differentiation of multipotent BMSCs into OBs mediated through the MAPK pathway. In addition, melatonin, betaine, and taurine also promoted OB formation by activating the ERK pathway.^73–75^ It is not difficult to conclude that most endogenous SMAs promote OB formation through the MAPK pathway. Taken together, studies have shown that the MAPK pathway regulates bone formation and that some SMAs exert a positive influence on bone formation by activating MAPKs. However, the overall net effect of the MAPK pathway on bone homeostasis is unclear because OC-mediated bone resorption can also be activated through MAPKs, which is further discussed in a subsequent section on OC pathways.

##### The Wnt signaling pathway

Wnt signaling is vital to bone homeostasis and affects almost all types of bone cells.^76^ β-Catenin is a central molecule of canonical Wnt signaling, and it forms a destruction complex with other proteins, including GSK-3β, APC, and Axin1/Axin2. Through stimulation by the Wnt ligand, the destruction complex proteins are dissociated, leading to the accumulation of β-catenin in the cytoplasm that is then translocated to the nucleus to enhance the expression of its downstream target genes (Fig. 4a).^77^ In contrast, the inhibition of Wnt leads to bone loss.^78^ Berberine enhanced the expression of β-catenin and further upregulated the expression of OB marker genes such as OPN and OCN, thereby increasing the MSC differentiation rate in vitro.^79^ Leonurine hydrochloride, a synthetic chemical compound with antioxidant and antiapoptotic activities derived from leonurine, was shown to promote phosphorylation of GSK-3β to enhance the activity of β-catenin, thereby accelerating bone formation.^80^ Similarly, OB differentiation was increased by stimulating the Wnt/β-catenin signaling pathway via treatment piperine treatment, resulting in elevated bone mineral density in OVX mice.^81^ In addition to alkaloid-SMAs, many endogenous SMAs (e.g., adenosine, Gln, taurine, glucosamine, and Sphingosine-1-phosphate) facilitated bone formation through the Wnt pathway. Studies have revealed that the promotion of OB formation via the Wnt signaling pathway relies on Gln metabolism. Specifically, Gln synthetase (Gls)-dependent Gln catabolism was necessary for Wnt-induced OB differentiation.^82^ Moreover, Gln promoted the growth, migration, and differentiation of hDPCs to accelerate pulp repair and regeneration by activating Wnt pathways.^70^ After treatment with 100 μmol·L^-1^ glutamate, the deamination of Gln increased ALP activity and extracellular matrix mineralization.^83^ Taurine, a nonessential amino acid in humans, was synthesized from the sulfur-containing amino acids methionine and cysteine.^84^ It suppressed the expression of inhibitors of Wnt signaling, such as sclerostin and DKK1 synthesized by OCs.^85^ In a low dosage regimen (1 μmol·L^−1^), Doxycycline, a broad-spectrum antibacterial drug, has been demonstrated to enhance the expression of β-catenin, Runx2, and OCN, thereby increasing osteogenic differentiation of MSCs derived from diabetic rats.^48^ Benidipine, an antihypertensive drug, upregulated the expression of Runx2, ALP, and OCN and activated Wnt/β-catenin signaling in vitro and in vivo, advancing bone formation.^86^ Metformin, the first-line drug for the treatment of type 2 diabetes, was confirmed to inhibit the phosphorylation of GSK-3β, increasing its activity, and increase the steady-state levels of the β-catenin protein, thus promoting the osteogenic differentiation of hBMSCs.^87^ In addition, 6-bromoindirubin-3′-oxime, an inhibitor of GSK-3β, also triggered the Wnt/β-catenin signaling pathway and enhanced the osteogenic differentiation of canine BMSCs.^88^ All kinds of SMAs facilitate OB differentiation by stimulating Wnt/β-catenin. These outcomes may be due to the extensive interactions between the Wnt/β-catenin pathway and other pathways and the multiple functions of Wnt/β-catenin signaling in various life activities of cells.

##### The PI3K/Akt signaling pathway

Phosphatidylinositol-3-kinases (PI3Ks) constitute a family of lipid kinases.^89^ The PI3K/Akt signaling pathway mainly affects cell metabolism, proliferation, migration, differentiation, and apoptosis.^90^ Growth factors or hypoxia, among other factors, can activate PI3K, leading to the phosphorylation of AKT, which triggers the activation of downstream mammalian target of rapamycin (mTOR) pathway, vascular endothelial growth factor (VEGF), etc., increasing MSC survival, proliferation, migration and angiogenesis (Fig. 4a).^90^ Leonurine, an alkaloid from *Herba leonuri*, enhanced the proliferation and differentiation of rat BMSCs administered at a 10 μmol·L^−1^ dose, and the effect was mediated through autophagy, which depended on the PI3K/AKT/mTOR pathway.^91^ Studying endogenous SMAs, Mirones et al.^92^ showed that dopamine enhanced the migration of mesenchymal progenitor cells via the PI3K/Akt pathway, which was suppressed by D2-class receptor antagonists or blocking antibodies. Glimepiride, an anti-type 2 diabetes drug, has been shown to facilitate the proliferation and differentiation of OBs through the PI3K/Akt pathway in rats.^93^ Adenosine, a natural nucleoside, is essential for all cellular life events because it is involved in energy production and utilization in the body. Its role in bone homeostasis has been widely recognized. For example, human osteoprogenitor cells are known to produce adenosine and express four adenosine receptor subtypes, namely, the A1 receptor, A2A receptor, A2B receptor, and A3 receptor.^94^ Gharibi et al.^95^ revealed that adenosine receptors, especially A2B, were expressed and activated during the differentiation of MSCs into OBs.^95^ In addition, both activation and overexpression of the A2B receptor promoted the expression of Runx2 and ALP in OBs, increasing the differentiation and mineralization rates of OBs and the formation of bone in vivo.^95,96^ The expression of the A2A receptor was upregulated in the late stage of OB differentiation.^95^ Furthermore, the Akt signaling pathway was activated and the nuclear localization of β-catenin was enhanced in MC3T3C-E1 and primary murine OBs treated with CGS21680, a highly selective A2A receptor agonist, promoting bone regeneration in vivo (Fig. 4c).^97^ In addition, studies have shown that S1P stimulates OB migration, prolongs their survival, and inhibits their apoptosis via the activation of the PI3K/Akt signaling pathway.^98–100^ Endo et al.^101^ demonstrated that the phosphorylation of Akt in the PI3K/Akt signaling pathway indirectly activated the Wnt/β-catenin pathway by mediating the inactivation of GSK-3β. Therefore, the PI3K/Akt pathway and Wnt/β-catenin pathway may exert synergistic effects on bone formation. For instance, both adenosine and sphingosine-1-phosphate promote OB formation by activating the PI3K/Akt and Wnt/β-catenin signaling pathways. Collectively, studies have indicated that various SMAs enhance bone formation via the PI3K/Akt signaling pathway.

##### The AMPK signaling pathway

AMPK controls the osteogenic differentiation of hMSCs through early mTOR inhibition-mediated autophagy and late activation of the Akt/mTOR signaling axis.^102^ Runx2, a novel substrate of AMPK, directly phosphorylates the serine 118 residue in the DNA-binding domain of Runx2.^103^ Adil et al.^104^ demonstrated that the expression of Runx2 increased through activation of the AMPK pathway upon oral administration of berberine (100 mg·kg^-1^) for 12 weeks in vivo. Similarly, piperine was shown to enhance OB differentiation through AMPK-dependent Runx2 expression in MC3T3-E1 cells.^105^ In addition, sanguinarine was identified as a candidate for use as an osteoporosis drug due to its induction of OB differentiation mediated via the AMPK/Smad1 signaling pathway and promotion of bone formation in a rat model of ovariectomy osteoporosis (OVX).^106^ In addition, it has been shown that metformin enhanced the osteogenesis of stem cells from human exfoliated deciduous teeth by activating the AMPK pathway (Fig. 4d).^107^ In particular, some SMAs have been identified as activators of the AMPK pathway by protecting OBs. For instance, OSU53 attenuated the damage to OBs induced by dexamethasone or glucose.^108^ Palmitate-induced apoptosis in bone marrow-derived osteoblastic cells has been proven to be impeded by AICAR treatment, which restored the activity of the ERK pathway via the activation of AMPK.^109^ A-769662 and GSK621 suppressed apoptosis or ameliorated the damage to OBs induced by H_2_O_2_ through the activation of AMPK.^110–112^ It seems that the AMPK pathway enhances bone formation mainly through its positive influence on Runx2 and protection of OBs. The SMAs that mainly stimulate the AMPK pathway are alkaloid-SMAs and activators (other-SMAs) in the AMPK pathway. However, activators of the AMPK pathway have not been experimentally confirmed in vivo, which may hinder their further development into drugs.

##### Other pathways

In addition to the aforementioned pathways, SMAs can ameliorate bone formation through other pathways, such as the HIF, mTOR, and BMP signaling pathways. OBs produce erythropoietin (EPO) in an HIF-dependent manner under physiological and pathophysiological conditions; in other words, OBs express HIF-1 and HIF-2, further activating the expression of EPO via increased transcription, thereby enhancing angiogenesis.^113^ Stegen et al.^114^ found that concurrent changes in Gln and glycogen metabolism, which depend on HIF1α, were vital to cell survival and led to increased bone formation. In addition, DFO promoted angiogenesis and osteogenesis by increasing the expression of HIF1α/VEGF.^115^ Regarding the mechanisms of action, HIF-1 is involved in redox regulation of bone homeostasis, which is explained in detail in a subsequent section.

The mTOR signaling pathway is of great importance to bone homeostasis because it regulates the proliferation of OBs and OCs.^116^ Glucosamine promoted the proliferation of OBs through the mTOR pathway, thereby promoting bone regeneration.^117,118^ Tranylcypromine, a small-molecule inhibitor of histone lysine-specific demethylase 1, has been proven to facilitate bone formation both in vitro and in vivo.^119^ It has also been reported that tranylcypromine affected osteogenesis through the BMP pathway and Wnt7b-mTORC1 signaling since both the mRNA and protein levels of BMP2 and Wnt7b were increased after treatment with 50 μmol·L^-1^ tranylcypromine for 48 h.^119^

The BMP pathway is considered a vital pathway in bone formation. Yonezawa et al.^120^ claimed that harmine-induced OB differentiation of MC3T3-E1 cells, primary calvarial OBs, and the C3H10T1/2 MSC lines by activating the BMP pathway and subsequently upregulating the gene expression of Runx2. Metformin has also been proven to enhance OB differentiation from MSCs obtained from type 2 diabetes mellitus (T2DM) samples through the BMP-4/Smad/Runx2 signaling pathway.^121^ In addition, γ-aminobutyric acid promoted the osteogenesis of MSCs through the upregulation of TNFAIP3.^122^ Wu et al.^123^ found that purmorphamine was a small-molecule agonist of Hedgehog signaling and induced osteogenesis in multipotent mesenchymal progenitor cells. In addition to promoting OB differentiation, some SMAs, such as Gln, increase the bone formation rate through energy metabolism, redox, and other pathways. Gln generates important reducing substances such as glutathione and the nutrient glutamate in the body, all of which participate in nutrient metabolism, redox and energy metabolism via multiple signaling pathways, including the Wnt, mTOR, and reactive oxygen species (ROS) signaling pathways, to promote cell proliferation, lineage distribution and bone formation^124^; notably, whereas Gln deficiency leads to decreased bone formation.^125^

Src family kinases are crucial targets in bone homeostasis. On the one hand, c-Src has been shown to increase the bone resorption rate in mice.^126^ On the other hand, the reduction in Src expression stimulates OB differentiation and bone formation.^127,128^ Dasatinib, a Src inhibitor, administer in low doses has been shown to promote osteogenic differentiation of MSCs obtained from multiple myeloma patients and healthy donors. Moreover, further experiments showed that it increased trabecular bone formation in vivo, which was primarily attributable to increased OB formation and activity rather than to an inhibitory effect on OC formation.^129^ In addition, dasatinib stimulated chondrogenic differentiation of MSCs via the Src/Hippo-YAP signaling pathway.^130^ Therefore, dasatinib may be a potential drug for bone diseases.

In fact, some other SMAs promote OB differentiation, such as tetramethylpyrazine,^131^ arecoline,^132^ and cinchonine.^133^ However, the underlying molecular mechanisms are unclear and need to be further elucidated.

#### Inhibition of OC activity

**Fig. 5:**
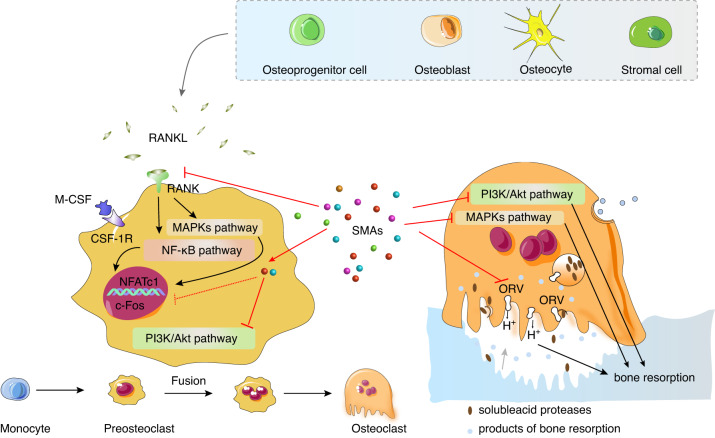
Mechanisms by which SMAs inhibit osteoclast (OC) formation. SMAs inhibit OC formation by regulating the NF-κB, MAPKs, PK13/ATK, ORV, and other signaling pathways. OCs are large multinucleated bone-resorbing cells derived from monocytes. The preosteoclasts (pre-OCs) generated by monocytes migrate to the bone surface and fuse into multinucleated OCs through the activation of RANKL and then develop into mature OCs. RANKL is generated by OBs, osteoprogenitor cells, osteocytes, and stromal cells. M-CSF binds to the transmembrane receptor CSF-1R expressed by pre-OCs to promote the proliferation and survival of the pre-OCs. The NF-κB pathway is mediated by the upstream RANKL-RANK complex, which leads to the differentiation of pre-OCs into OCs via the activation of downstream signaling molecules such as NF-κB, c-Fos, and NFATc1. OC ruffled-border H+-ATPases (ORVs) emerge at the OC ruffled border and display novel functions in OCs to solubilize bone minerals by acidifying an extracellular resorption compartment

OCs are critical for bone resorption; therefore, inhibiting the formation and activity of OCs is beneficial for reducing bone loss and maintaining bone homeostasis. Some possible mechanisms for the influence of SMAs on OCs are described (Fig. 5).

##### The MAPK signaling pathway

The stimulation of the MAPK signaling pathway activates OC differentiation (Fig. 5). The MAPK signaling pathway can be stimulated by the upstream RANKL-RANK interaction.^134^ Matsumoto et al.^135^ discovered that the p38/MAPK signaling pathway was necessary for OC formation induced by RANKL. Many alkaloid-SMAs alleviate bone resorption by inhibiting MAPK signaling pathway activation. Vinpocetine suppressed the phosphorylation of ERK and JNK involved in osteoclastogenesis and attenuated OVX-induced bone loss in vivo.^33^ Li et al. revealed that sanguinarine treatment impeded OC formation and bone resorption.^136^ In addition, piperine was shown to hinder OC differentiation by suppressing p38/NFATc1/c-signaling.^137^ Deferoxamine ameliorated bone loss by suppressing OC differentiation partially through MAPK signaling.^138^ Moreover, norisoboldine,^139^ Cytisine,^140^ and L-tetrahydropalmatine^141^ inhibited MAPK pathway activation to prevent OC formation. However, as previously mentioned, MAPKs, such as ERK1 and ERK2,^142^ also exert positive effects on OB differentiation. Herein, the positive or negative effects of SMAs that regulate bone homeostasis through the MAPK pathway need to be verified in vivo under different conditions. Notably, we found that most alkaloid-SMAs regulated OC activity via the MAPK pathway, while most endogenous-SMAs regulated OB differentiation via the MAPK pathway. Thus, alkaloid-SMAs and endogenous-SMAs may effectively alter bone homeostasis through the MAPK pathway in different cell types.

##### The NF-­κB signaling pathway

The NF-κB family consists of five protein monomers, including p50, p52, RelA (p65), c-Rel, and RelB, and they form homodimers or heterodimers that differentially bind DNA.^143^ The NF-κB signaling pathway is essential for OC formation and bone resorption (Fig. 5). The NF-κB pathway is mediated through an upstream RANKL-RANK combination, which activates downstream signaling such as NF-κB, c-Fos, and NFATc1 signaling,^144–149^ contributing to inhibited osteogenic differentiation of BMSCs and differentiated pre-OCs into OCs. Moreover, Yamashita et al. confirmed that NF-κB p50 and p52 regulated receptor activator of NF-κB ligand (RANKL) by activating c-Fos and NFATc1.^150^ Additionally, inhibition of NF-κB also hampered inflammation in bone diseases, which is explained in detail in a subsequent section. Hence, the inhibition of NF-κB signaling is conducive to bone regeneration.

Among SMAs, most alkaloid-SMAs have been shown to inhibit OC formation or exert anti-inflammatory effects through the NF-κB pathway, improving bone homeostasis. For instance, nuciferine, derived from lotus, inhibited OC formation by decreasing the expression of OC-specific genes and proteins via the inhibition of MAPK and NF-κB pathway activation.^151^ Furthermore, it promoted type H vessel formation^151^ to ameliorate bone loss caused by ovariectomy or breast cancer in vivo.^151,152^ Neferine, also isolated from *Nelumbo nucifera* (lotus), exhibits anti-inflammatory and antioxidant properties, and recently, it was verified to suppress osteoclastogenesis and attenuate OVX-induced osteoporosis in vivo by inhibiting NF-κB pathway activation.^153^ Tetramethylpyrazine, one of the effective ingredients of the traditional Chinese medicine Ligusticum chuanxiong, with anti-inflammatory and antioxidant properties, activated the autophagy of MSCs derived from rats with glucocorticoid-induced osteoporosis (GIOP) to protect cells against apoptosis^25^ and reduced RANKL and IL-6 levels to inhibit osteoclastogenesis, thereby promoting osteogenesis and increasing bone mass in the GIOP state.^131^ Hu et al.^154^ discovered that tomatidine prevented OVX-induced bone loss in vivo. At the molecular level, in the presence of tomatidine, RANK-TRAF6 binding was abrogated, downregulating RANKL-induced JNK, p38, NF-κB, and Akt phosphorylation, resulting in the suppression of osteoclastogenesis.^154^ In addition, it has been reported that other alkaloid-SMAs, such as neotuberostemonine,^155^ tetrandrine,^156–158^ sanguinarine,^106,136^ vinpocetine,^33^ and norisoboldine,^139,159^ prevented OC formation via the NF-κB pathway. Notably, alkaloid-SMAs exert anti-inflammatory and antioxidant effects simultaneously and exhibit increased bone regeneration rates through the inhibition of bone resorption, which is discussed in a subsequent section. In addition, benzydamine, an anti-inflammatory drug, retarded the degradation of IκB kinase to inhibit the activation of NF-κB, attenuating bone loss in lipopolysaccharide- and OVX-treated mice.^45^ In addition, it has been proven that RANKL activation of NF-κB and MAPK pathways in bone marrow-derived macrophages (BMMs) was inhibited after cell treatment with meclizine (20 μmol·L^−1^).^160^ For endogenous SMAs, spermidine and spermine exerted negative regulation on the transcriptional activity of NF-κB in OCs in vitro and prevented OVX-induced bone loss.^161^ The inhibitory effects of SMAs on OC activity mediated through the NF-κB pathway may be related to their alkalinity. However, no studies exploring the relationship between the alkalinity of SMAs and the inhibition of OC activity have been reported, and this connection deserves further study. On the basis of Table 3, we calculated that 97.05% of alkaloid-SMAs inhibited OC activity, and 63.64% of these SMAs suppressed OC activity by inhibiting NF-κB pathway activation.

##### The PI3K/Akt signaling pathway

The PI3K/Akt signaling pathway positively affects OBs according to the aforementioned studies, and it exerts a similar effect on OCs.^162^ For example, some SMAs suppress bone resorption by inhibiting the PI3K/Akt pathway and AKT activation by regulating the GSK3β/NFATc1 signaling cascade in pre-OCs or Ocs.^163^ Zhong et al.^158^ demonstrated that injecting tetrandrine into mice after OVX markedly reduced bone loss. The effects of four different concentrations, 0.125, 0.25, 0.5, and 1 μmol·L^-1^ tetrandrine, were analyzed in the study. The results further showed that tetrandrine inhibited OC differentiation by suppressing the NF-κB, Ca^2+^, PI3K/AKT, and MAPK signaling pathways in BMMs and RAW264.7 cells in a dose-dependent manner. In addition, stachydrine has also been reported to prevent LPS-induced bone loss via NF-κB and Akt signaling. Notably, it inhibited osteoclastogenesis by suppressing RANKL-induced phosphorylation of Akt and GSK3β.^164^ SC79, an SMA, was found to activate Akt and downstream Nrf2 signaling in OBs, thereby protecting OBs from dexamethasone-induced oxidative stress.^165^ Another study reported that SC79 released from porous SC79-loaded ZSM-5/chitosan scaffolds enhanced the proliferation and osteogenic differentiation of hBMSCs, and results of an in vivo study further showed that it promoted new bone formation in cranial defects.^166^ Notably, inhibition of the PI3K/Akt pathway might exert negative effects on OBs. Therefore, although SMA inhibits the PI3K/Akt pathway in OCs, whether it exerts a negative effect on OBs needs to be determined. Cytisine and diaporisoindole E suppressed OC formation by inhibiting activation of the RANKL-induced PI3K-AKT signaling pathways without affecting OB differentiation in vitro.^140,167^ In contrast, it has been reported that cinchonine not only inhibited osteoclastogenesis through the AKT pathway but also enhanced OB differentiation,^133^ implying that mechanisms in addition to the PI3K/AKT pathway mechanism may play a major role in promoting OB differentiation. Overall, the role of the PI3K/Akt pathway in bone hemostasis remains unclear. Therefore, in vivo experiments are urgently needed.

##### The OC ruffled-border vacuolar H^+^-ATPase

Vacuolar H^+^-ATPases are vital ATP-dependent proton pumps, known as housekeeping enzymes in eukaryotic cells.^168^ In addition, the specific isoenzymes, OC ruffled-border H^+^-ATPases (ORV), emerge at the OC ruffled border and display specific functions in OCs; for example, they solubilize bone mineral by acidifying an extracellular resorption compartment.^169,170^ Specifically, ORV leads to lacunar acidification through proton pumping and soluble acid protease (e.g., cathepsin K, MMP9) release, which causes bone resorption (Fig. 5).^170^ Thus, the development of anti-bone-resorption drugs that function by inhibiting ORV has become a new strategy that has attracted attention. There are also several SMAs inhibit ORV and show the potential to be developed into anti-bone-resorption drugs. For instance, SB242784, a selective inhibitor of ORV, has been found to inhibit retinoid-induced hypercalcemia in thyroparathyroidectomized rats and bone loss in ovariectomized rats.^26,171^ The benzamide derivatives FR167356, FR202126, and FR177995 have also been reported to prohibit ORV in OCs and exert anti-bone-resorption effects.^172^ Moreover, KM91104, a benzohydrazide derivative, was shown to be s an effective molecule in terms of its inhibition of ORV to impede bone resorption.^173^ Enoxacin, a fluoroquinolone antibiotic, interfered with OC formation and activity, as evidenced by enoxacin inhibition of the differentiation of primary marrow cells and RAW 264.7 cells into OCs.^174^ In summary, ORV is a promising target for the treatment of bone loss diseases, and there therefore, its inhibitors show enormous potential to be developed into new drugs; however, the effects of ORV-targeting drugs need to be confirmed because evidence based on in vivo studies is rare.

##### Other signaling pathways

There are other ways for SMAs to inhibit OC formation. OCs need to take up and store Fe^2+^ to meet the increased energy demand during OC differentiation and bone resorption. DFO chelates Fe^2+^, thus inhibiting OC formation.^115^ In addition, metformin suppresses bone resorption by activating the AMPK pathway.^175^ It has been shown that dopamine suppressed OC differentiation in a D2-like receptor (D2R)-dependent manner. The binding of dopamine to D2R downregulated the cyclic adenosine monophosphate (cAMP)/protein kinase A (PKA) signaling pathway during osteoclastogenesis, resulting in decreases in cAMP-response element-binding protein (CREB) phosphorylation, and blocking D2R abolished the inhibitory effects of dopamine.^23^ Another neurotransmitter, epinephrine, has been proven to promote osteoblast differentiation by enhancing BMP signaling through a cAMP/protein kinase A signaling pathway.^176^ As mentioned above, Scr signaling inhibited bone resorption and promoted bone regeneration. Studies have shown that saracatinib reduced the formation of active phosphorylated c-Src in OC-like cells and reversibly prevented OC precursor migration from the OB layer to the bone surface and subsequent formation of actin rings and resorption pits.^41^

### Regulation of bone microenvironments

**Fig. 6:**
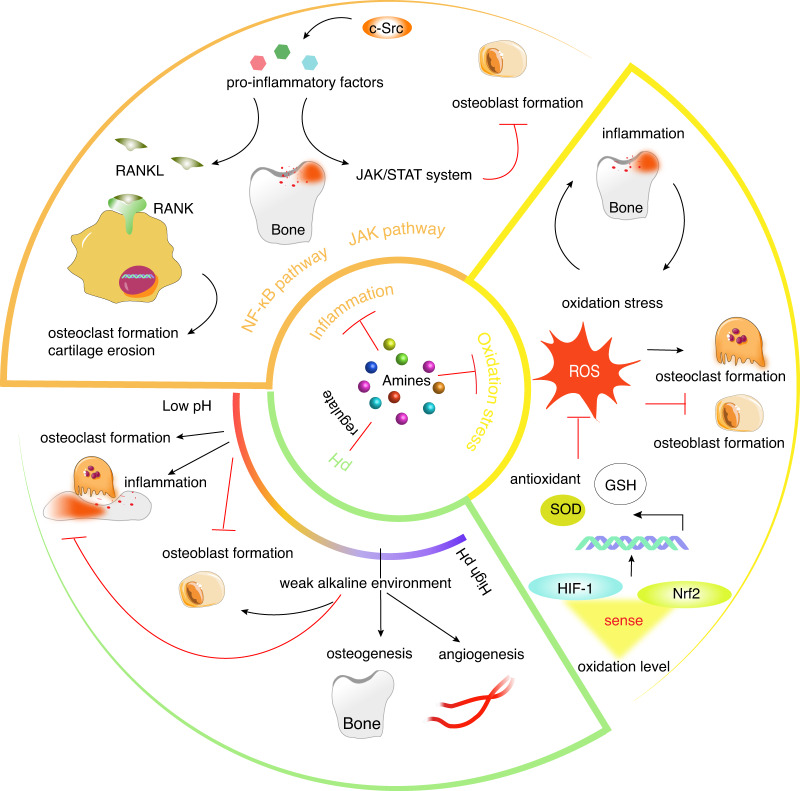
SMAs regulate bone homeostasis by influencing microenvironment characteristics including inflammation, oxidation stress, and pH. SMAs exert anti-inflammatory functions through the NF-κB pathway and JAK pathway. Some SMAs exert osteotropic activity by inhibiting oxidative stress through redox-related signaling such as HIF-1 or Nrf2 or downregulating the levels of ROS. The acid–base microenvironment plays an important role in bone regeneration. A weakly alkaline microenvironment can not only enhance osteogenesis and angiogenesis but can also suppress osteoclast formation and inflammation. However, a low pH microenvironment aggravates bone resorption and inflammation, prevents mineralization, and inhibits the formation of osteoblasts, leading to significant bone loss

SMAs play significant roles in the regulation of inflammation, oxidative stress, and pH on bone homeostasis (Fig. 6), in addition to their direct effects on OBs and OCs. Some SMAs exhibit anti-inflammatory, antioxidation, and alkaline properties, improving the bone microenvironment and thus contributing to bone homeostasis.

#### Anti-inflammation

Inflammation influences the progress of bone regeneration in bone diseases. Chronic inflammation can impede bone regeneration and cause other diseases such as RA, OA, diabetes mellitus, and systemic lupus erythematosus.^177^ Some SMAs exhibit anti-inflammatory functions mediated through the NF-κB and JAK pathways, regulating the behaviors of chondrocytes, OCs, or OBs in bone regeneration.

The NF-κB signaling pathway is not only a vital signaling pathway in OC formation but also a typical inflammation pathway. The majority of proinflammatory factors, including IL-1, TNF and IL-6, can increase the production of RANKL, leading to accelerated OC formation.^178–180^ Moreover, the activation of the NF-κB pathway also causes extracellular matrix (ECM) damage and cartilage erosion in response to the inflammation of chondrocytes in OA.^181^ Therefore, the regulatory effects of the NF-κB pathway on inflammation make it an important target for the treatment of inflammatory bone diseases.^182^ Some drug-SMAs with anti-inflammatory functions tend to inhibit OC formation via the NF-κB signaling pathway. For example, benzydamine suppressed IL-1β expression via the inhibition of NF-κB and AP-1, thereby inhibiting OC differentiation and bone resorption, and its inhibitory effects were reversed by IL-1β treatment.^45^ Furthermore, LPS- or OVX-induced bone destruction was ameliorated by the treatment of benzydamine at a dose of 10 mg·kg^-1^.^45^ Evidence showed that TNF was the major factor involved in the response to synovial arthritis in the OA context.^183^ In TNF-α-induced mouse models of OA, synovial inflammation was inhibited after spermidine treatment.^184^ Mechanistically, spermidine prevented TNF-induced NF-κB/p65 activation by suppressing RIP1 ubiquitination, thereby ameliorating cartilage degeneration and osteophyte formation.^184^ In addition, many alkaloid-SMAs exhibit anti-inflammatory effects by inhibiting the NF-κB pathway. For instance, tetramethylpyrazine has been reported to attenuate the senescent phenotype of cells and contribute to an anti-inflammatory and angiogenic microenvironment.^185^ Subsequently, an investigation performed by Yu et al. revealed that treatment with tetramethylpyrazine downregulated the inflammatory reaction of chondrocytes in the joint effusion of OA patients and alleviated the injury and matrix degradation induced by IL-1β.^186^ Mechanistically, it blocked NF-κB pathway activation, enhanced the expression of SOX9 and decreased the production of ROS in IL-1β-induced chondrocytes, thereby protecting chondrocytes.^186^ In addition, Duan et al. discovered that the inflammation and apoptosis of LPS-stimulated human periodontal ligament cells (PDLSCs) were reduced via the downregulation of miR-302b by tetramethylpyrazine.^187^ Thus, tetramethylpyrazine may be a potential drug for treating OA. Moreover, other alkaloid SMAs, such as tomatidine,^154^ tetrandrine,^157^ berberine,^188^ vinpocetine,^33^ dauricine,^189^ and nitensidine A,^190^ exert anti-inflammatory effects, which may contribute to a reduction in bone loss and an improved bone microenvironment.

Drug-SMAs, baricitinib and tofacitinib have been used for the treatment of inflammatory diseases such as RA.^191,192^ Recent research revealed that these two drugs, both SMAs, mitigated OVX-induced bone loss and inhibited bone loss in the context of arthritis in vivo.^27^

Some SMAs exert anti-inflammatory effects mediated via other pathways in addition to those mediated by the NF-κB pathway. For example, when bone was in an inflammatory condition, the inhibition of c-Src downregulated IL-6 expression.^128^ Therefore, c-Src inhibitors such as saracatinib might show osteotropic activity by suppressing inflammation.^42^ Moreover, another c-Src inhibitor, dasatinib, has been reported to inhibit inflammation and increase bone mineral density in a mouse model of chronic recurrent multifocal osteomyelitis.^193^ As mentioned above, adenosine exerts positive effects on bone regeneration. In addition, it is critical to reducing inflammation. However, adenosine has an extremely short half-life, which means it is rapidly metabolized in blood, where it is converted to other molecular forms.^194^ Adenosine N1-oxide, a product of adenosine oxidation at the N1 position of the adenine base moiety, has been found in royal jelly, and it was more stable than adenosine and maintained anti-inflammatory activity.^195^ Specifically, it inhibited the secretion of TNF-α and IL-6 in LPS-treated RAW264.7 cells via its action on the PI3K/Akt/GSK-3β pathway, further promoting the osteogenic differentiation of MC3T3-E1 cells.^195^ Additionally, cetirizine, a histamine 1 receptor antagonist, promoted bone formation after suture expansion, mostly by suppressing osteoclastic activity.^46^ According to the aforementioned studies, SMAs with anti-inflammatory effects improve bone homeostasis, although their mechanisms of action may differ.

#### Antioxidation

Oxidative stress, caused by high levels of ROS, increases osteoclastogenesis and inhibits osteogenesis and mineralization by inducing the apoptosis of OBs and OCs, leading to dysfunctional bone.^196^ Thus, inhibition of oxidative stress can be a feasible strategy for treating bone diseases. It has been reported that certain SMAs inhibit oxidation stress through redox-related signaling factors such as hypoxia-inducible 1 (HIF-1) or the Nrf2 pathway in bone homeostasis. In addition, oxidative stress is associated with inflammation (Fig. 5). ROS can lead to the generation of proinflammatory molecules, inducible NO synthase (iNOS) and cyclooxygenase (COX-2),^197^ and inflammation also causes the increased production of ROS. Hence, the regulation of oxidation levels in cells or tissues is crucial for bone homeostasis.

HIF or Nrf2 proteins can be rapidly stabilized by hypoxia or oxidative stress, respectively, to respond to induce rapid changes in the redox state of cells,^198^ thereby protecting cells. HIF-1, composed of the HIF-1α subunit and HIF-1β subunit, among which HIF-1α is the core of the oxygen sensing mechanism,^199^ regulates the expression of many antioxidants.^200–202^ Jing et al. found that DFO upregulated HIF-1α expression in a dose-dependent manner, and the downregulated expression of HIF-1α induced by dexamethasone was rescued by DFO treatment (100 μmol·L^-1^).^115^ Similarly, the activation of Nrf2 promoted the transcription of antioxidant enzymes (e.g., SOD) and the production of antioxidant substances (e.g., GSH).^203^ Lack of Nrf2 induced oxidative stress and promoted OC differentiation induced by RANKL, resulting in bone loss.^204^ Chen et al. declared that the inhibition of Nrf2 resulting from aberrant DNA methyltransferase level elevation and subsequent Nrf2 promoter hypermethylation was probably a vital epigenetic mechanism underlying the pathogenesis of osteoporosis.^205^ In addition, Nrf2 reduced the toxicity of iron-induced oxidative stress.^198^ For example, Jia et al.^206^ showed that metformin ameliorated oxidative stress caused by H_2_O_2_ in PDLSCs, and pretreatment or cotreatment with metformin reversed the activity of SOD and the concentrations of GSH and ROS, thereby protecting cells from oxidative stress. Moreover, the positive effect of metformin on cell viability was diminished by the knockdown of Nrf2.^206^

Notably, some agonists of the AMPK pathway, including OSU53,^108^ AICAR,^109^ A-769662,^110^ GSK621,^111^, and Compound 13,^207^ are antioxidants and protect OBs from oxidative damage, which might be related to the relationship between AMPK and Nrf2. Joo et al. discovered that a subnetwork integrating neighboring molecules suggested a direct interaction between AMPK and Nrf2.^208^ They found that AMPK stimulation caused nuclear accumulation of Nrf2 and that AMPK phosphorylated Nrf2 at the Ser558 residue (Ser^550^ in mice) located in the canonical nuclear export signal peptide.^208^

Additionally, SMAs can influence other signals by regulating oxidative stress. For instance, it has been reported that dauricine decreased oxidation of serine/threonine-protein phosphatase 2A to block the activation of NF-κB by reducing the ROS levels in OCs, resulting in protection from bone loss.^189^ Zhan et al.^209^ demonstrated that treatment with vindoline suppressed intracellular ROS production in a dose-dependent manner, thus inhibiting OC differentiation. Similarly, Zhu et al. revealed that vinpocetine inhibited the RANKL-induced production of ROS and increased the expression of cytoprotective enzymes such as HO-1 and NQO-1.^33^ In addition, in IL-1β-treated chondrocytes, the level of SOD was significantly decreased, and the production of ROS and MDA was increased, which was attenuated by treatment with ligustrazine.^186^ Pyrroloquinoline quinone (PQQ), a powerful antioxidant, has been shown to prevent bone loss in mice after orchiectomy (ORX).^210^ Mechanistically, PQQ reduced the elevated ROS levels in thymus tissues and partially promoted the expression of antioxidant enzymes such as SOD-1 and SOD-2 in the mice after ORX.^210^ Taken together, the studies show that SMAs with antioxidant effects protect OBs or chondrocytes from oxidative stress or inhibit osteoclastogenesis by interacting with various pathways. In conclusion, SMAs with antioxidant effects are endogenous SMAs, alkaloid-SMAs, anti-inflammatory drug SMAs, and AMPK pathway activators.

#### Influence of local pH

The acid–base microenvironment, which affects the proliferation, differentiation, and apoptosis of bone tissue-related cells, is being increasingly appreciated. Acidosis not only accelerates bone resorption and bone mineral dissolution but also prevents mineralization and OB formation, leading to severe bone loss.^211–213^ In contrast, a weakly alkaline microenvironment can promote bone formation/mineralization^214^ and reduce bone resorption.^215^ Even subtle changes in the extracellular pH can affect the secretion of phenotype-inducing proteins in OBs, including collagen and osteocalcin.^216^ ALP activity and collagen synthesis were found to be increased by 2–3 fold when the pH value was from 6.6 to 7.8.^216^ Weak alkalinity increased the proliferation and differentiation of OBs.^217^ In addition, the acid–base microenvironment can also regulate inflammation and blood vessel formation. It has been reported that an acidic microenvironment increases the expression of certain inflammatory factors, such as IL-6 and cathepsin B, in a time-dependent manner.^218^ Spector et al.^219^ discovered that compared with a neutral environment (pH = 7.4), an acidic environment (pH = 7.0) decreased the production of VEGF in OBs. Overall, the acid–base microenvironment contributes to an alkaline microenvironment, which is conducive to bone formation.

Recent findings suggest that creating an alkaline microenvironment by releasing alkaline ions from tissue-engineered materials has great benefits for bone regeneration.^220^ It has been confirmed that the differentiation potential and pit-formation capability of OCs were greatly suppressed when they were cultured in titrated material extracts with a pH value of 7.8 or higher.^221^ Moreover, Liu et al. revealed that the expression of OC-related enzyme genes, including cathepsin K, TRAP, MMP-9, and NFATc1, was inhibited under alkaline conditions, with a pH 7.8−8.0, in all tested materials.^221^ Therefore, providing a relatively alkaline microenvironment on the surface of biodegradable implant material may be a great strategy to inhibit the activity of OCs and thus promote bone regeneration.^221^ Our groups have performed experiments with variations in the interfacial pH values of poly (D, L-lactide) (PDLLA) and P-PUUs. The results demonstrated the interfacial pH rapidly decreased after the release of degraded products, and this outcome was reversed by the introduction of the alkaline segments of piperazine.^222^ More intriguingly, OBs also constructed the microenvironment by secreting cellular metabolites, including ALP and extracellular calcium, to upregulate the interfacial pH of the materials, thereby promoting their own proliferation, differentiation, and mineralization.^222^ Moreover, compared with PDLLA, P-PUU showed a greater ability to promote OB differentiation, which is attributed to the piperazine units in the P-PUU.^222^ Hence, the addition of proper alkaline molecules or units to biodegradable biomaterials can create a weak alkaline microenvironment, which is beneficial to bone regeneration. SMAs may be considered candidates for loading onto bone tissue-engineered materials due to their alkaline properties. The investigations into the relationship between local pH values and the behaviors of OCs and OBs demonstrated that an alkaline microenvironment inhibited OC activity and enhanced OB formation. However, they mainly focused on the local pH of the biomaterials and did not specifically explore the specific relationship between SMA alkalinity and bone homeostasis.

## Current challenges and prospects

### Challenges to the development of SMAs to treat bone diseases

In recent years, SMAs have been developed as potential drug molecules or biomaterials with broad applications for the treatment of bone diseases. However, large challenges for SMA development remain, and each needs to be further investigated.

For the development of SMA-based drugs, the main challenges are summarized as follows:

1. Some studies on SMAs for bone homeostasis lack validation via in vivo experiments. The in vivo effect of SMAs may be very different from that observed under in vitro conditions. Therefore, when only in vitro studies are carried out, the regulatory effects of SMAs on bone homeostasis are still unconvincing.
2. The mechanisms by which SMAs enter cells and interact with receptor proteins are still unclear. Most studies have led to the identification of only one or two signaling pathways. More comprehensive studies are still needed.
3. SMAs have been shown to exert antioxidative and anti-inflammatory effects. However, the detailed mechanisms of these effects have not been clearly characterized.

For the development of SMA-based biomaterials, the main challenges are summarized as follows:

1. The stability and reproducibility of a synthetic process for producing SMA-based biomaterials are insufficient and need to be further optimized. On the one hand, the most recently available biomaterials lack standardized preparation procedures or evaluation criteria. As a result, the reproducibility of biomaterial experiments is not sufficiently high. On the other hand, the successful loading SMAs onto biomaterials heavily depends on the physical and chemical properties of the SMAs. We need to develop a proper design to load SMAs with different chemical structures, such as RNH_2_, RNH, and R_3_N SMAs.
2. There is a lack of in vivo data about the local release of SMAs from SMA-based biomaterials. The release mechanisms and degradation kinetics for most SMA-based biomaterials have not been clearly determined. More work is needed to further improve SMA loading efficiency at effective and safe concentrations.

### Prospects

#### SMA-based drug development

To address the above problems and further promote the development of SMA-based drugs, the following points should be emphasized:

1. The extensive screening of SMAs with osteotropic activity should be increased. Effective SMAs will be identified from libraries of traditional Chinese medicine by new technologies such as network pharmacology and high-throughput omics, and the efficacy of positive molecules needs to be compared with that of the most commonly used drugs.
2. In vivo, efficacy should be used as the primary criterion for SMA-drug evaluation before further mechanistic studies are performed.
3. The underlying mechanisms of SMA effects on the maintenance of bone homeostasis need to be comprehensively and precisely understood. New techniques, such as single-cell RNA sequencing, proteomic profiling, differential expression analysis, and pathway analysis, may be applied to achieve this level of comprehension.^223^ The SILAC + SM pull-down technique has been used to determine the specific binding of small molecules to proteins,^224^ and it can be used in future studies.
4. The relationship between the chemical structure and biochemical activity of SMAs needs to be further explored, which may provide robust guidance for their in vivo application. For example, in-depth structure–activity studies of SMAs anti-inflammation, antioxidation and pH-alter effects may lay a solid foundation for the development of SMA-based drugs and biomaterials.

#### SMA-based biomaterials development

Some prospects for SMA-based biomaterial development should also be emphasized:

1. Establishing an SMA-based biomaterial with a stable and reproducible synthesis process is urgent for its scalable production. The synthesis of SMA-based biomaterials that is stable and reproducibility should be identified, and effective loading strategies for similar structures and properties of SMAs need to be classified. Moreover, the selection of a biomaterial with proven preparation methods is crucial for enhancing the stability and reproducibility for SMA-based biomaterial synthesis.
2. Developing an SMA controlled-release strategy is important for SMA-based biomaterials. Hydrogels, fibrous structure biomaterials, porous microspheres, etc., may be applied to respond to the pH, temperature, and oxidation conditions of a microenvironment. Micro/nanorobots are promising drug-targeted delivery systems, and 3D printing techniques can achieve precise loading and controlled release of SMAs. In addition, a combination of in vitro and in vivo methods should be developed to prepare SMA-based biomaterials with the desired drug-loading procedure and sustained release period.
3. Selecting SMAs with definitive treatment effects or biomaterials that have received approval for clinical use can reduce the R&D time. Mechanistic studies can further promote the development of SMA-based biomaterials.
